# Zerumbone ameliorates high-fat diet-induced adiposity by restoring AMPK-regulated lipogenesis and microRNA-146b/SIRT1-mediated adipogenesis

**DOI:** 10.18632/oncotarget.16974

**Published:** 2017-04-08

**Authors:** Jiyun Ahn, Hyunjung Lee, Chang Hwa Jung, Won Hee Choi, Tae Youl Ha

**Affiliations:** ^1^ Research Group of Metabolic Mechanism, Korea Food Research Institute, Seongnam, Korea; ^2^ Division of Food Biotechnology, University of Science & Technology, Daejeon, Korea

**Keywords:** obesity, zerumbone, SIRT1, microRNA-146b, adipogenesis, Pathology Section

## Abstract

Obesity is characterized by increased fat mass, as adipose tissue serves as a storage site for excess energy from food consumption. In obesity, altered lipid metabolism of adipose tissue, characterized by fatty acid uptake, de novo lipogenesis, and lipolysis, are induced. In this study, we examined the effect of zerumbone, a major sesquiterpene from wild ginger, on high-fat diet (HF)-induced obesity and dysregulated lipid metabolism in the white adipose tissues (WAT) of C57BL/6N mice. Dietary supplementation with zerumbone ameliorated HF-induced obesity and improved impaired lipid metabolism in WAT. Zerumbone additionally induced AMPK activation and phosphorylation of acetyl-CoA carboxylase, and effectively decreased adipogenic differentiation, in a concentration-dependent manner in the 3T3-L1 cells. Dysregulated microRNAs in obese WAT and adipocytes were examined, and zerumbone treatment was found to effectively reverse the robust upregulation of microRNA-146b. An increase in the levels of SIRT1, the direct target of microRNA-146b, was observed in zerumbone-treated differentiated adipocytes. This increase was additionally observed in WAT of zerumbone-supplemented mice. The antiadipogenic effect of zerumbone was found to be abolished in SIRT1-silenced 3T3-L1 cells. The increase in SIRT1 levels induced by zerumbone led to deacetylation of FOXO1 and PGC1α in WAT and differentiated 3T3-L1 cells. These findings indicate that zerumbone ameliorated diet-induced obesity and inhibited adipogenesis, and that the underlying mechanisms involved AMPK and the microRNA-146b/SIRT1 pathway. Zerumbone may represent a potential therapeutic candidate for the prevention and treatment of metabolic diseases, particularly obesity.

## INTRODUCTION

Obesity is an energy imbalance disorder in which nutrient intake chronically exceeds energy expenditure, resulting in the increased fat mass [1]. Obesity is the primary risk factor for metabolic syndrome, which is characterized by hypertension, type 2 diabetes, and cardiovascular disease [2]. The worldwide prevalence of obesity has more than doubled since 1980. According to WHO statistics, 39% of adults over 18 years old were overweight, and 13% were obese in 2014. Unless the current trend is reversed, the continuing increase in obesity will have detrimental effects on global health and economy. Despite advances in weight management strategies in recent years, the treatment of obesity remains a significant challenge. The potential of natural phytochemicals as substitutes for weight management [3] and their ability to increase fatty acid β-oxidation, fat absorption, and suppress appetite [4] have received much interest.

Zerumbone, a cyclic sesquiterpene present in rhizomes of the wild ginger *Zingiber zerumbet* Smith, has been identified as a tumor suppressor [5, 6] in various cancer cells. Zerumbone induces cell cycle arrest [7], inhibits TGF-β1 signaling [8, 9], and inhibits tumor angiogenesis by blocking NF-κB signaling [10]. In addition, zerumbone exhibits a wide range of pharmacological activities such as anti-cataractogenesis [11], anti-inflammatory effects [12], and anti-viral effects [13]. Zerumbone has also been shown to attenuate nonalcoholic fatty liver [14], mitigate hypertriglyceridemia [15], and ameliorate diabetic nephropathy [16]. However, the effect of zerumbone on obesity has not been reported to date.

MicroRNAs (miRNAs) are highly conserved, small non-coding RNAs that regulate gene expression at the post-transcriptional level by binding to complementary sites on their target transcripts and either inducing mRNA cleavage or repressing protein translation [17]. The dysregulation of miRNA is important in cancer initiation and progression [18]. The miRNAs have emerged as important post-transcriptional regulators of adipocyte metabolism, energy homeostasis, lipid metabolism, pancreatic β-cell development, and weight gain resulting from a high-fat diet [19–22]. Among them, miR-27a and miR-27b have been reported as negative regulators of adipocyte differentiation through peroxisome proliferator-activated receptor gamma (PPARγ) inhibition [23]. Natural compounds such as persimmon tannin have been shown to inhibit the differentiation of 3T3-L1 cells through upregulation of miR-27a and miR-27b [24]. We previously reported that miR-146b functions as a positive regulator of adipocyte differentiation through downregulation of sirtuin 1 (SIRT1) [25]. However, chemicals from natural sources that could downregulate miR-146b were not investigated.

In this study, we examined the effect of zerumbone on high-fat diet-induced obesity and measured the expression levels of lipid metabolism-regulated genes in adipose tissue in mice and differentiated 3T3-L1 cells. We also measured the effect of zerumbone on 5’ AMP-activated protein kinase (AMPK) phosphorylation. To investigate the role of miRNA in the anti-obesity effects of zerumbone, we analyzed miRNA expression profiles in adipose tissue and performed a functional study to validate miRNA-target mRNA interactions.

## RESULTS

### Zerumbone decreased adiposity in C57BL/6N mice with diet-induced obesity

**Figure 1:**
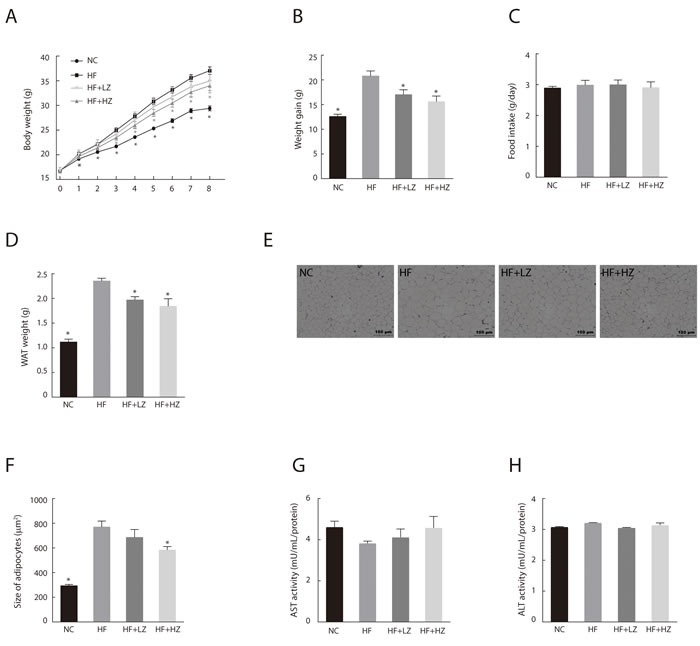
Zerumbone ameliorated diet-induced adiposity in C57BL/6N mice **A**. Body weight of mice from the NC, HF, HF + LZ, and HF + HZ groups was measured weekly during the experimental period (*n* = 10). NC, mice fed normal control diet; HF, mice fed high-fat diet; HF+LZ, mice fed high-fat diet containing 0.01% zerumbone; HF+HZ, mice fed high-fat diet containing 0.025% zerumbone. **B**. Effect of zerumbone on body weight gain after 8 weeks of experimental diet. **C**. Measurement of food intake during experimental period. **D**. Effect of zerumbone on epididymal white adipose tissue (WAT) weight. **E**. Histological staining of WAT. Epididymal adipose tissues were isolated and stained with H&E and examined microscopically. **F**. Average adipocyte size was quantified in H&E-stained paraffin sections in five mice per group. *P < 0.05 versus HF. **G**. Aspartate aminotransferase (AST) and **H**. alanine transaminase (ALT) activity were measured in liver homogenates.

To determine the effect of zerumbone on obesity, C57BL/6N mice were fed a high-fat diet (HF) containing 0.01% (HF + LZ) or 0.025% (HF + HZ) zerumbone for 8 weeks (Figure 1A). Mice fed a normal control diet (NC) gained 12.59 ± 0.46 g, whereas the HF-fed mice showed a significantly higher body weight gain of 20.81 ± 1.01 g (Figure 1B). However, HF + LZ and HF + HZ resulted in lower weight gain (18.2% and 25.1%, respectively) than that in the HF group (*P* < 0.05). Food intake did not differ between the groups (Figure 1C). The weight of epididymal white adipose tissue (WAT) was significantly reduced in the zerumbone-supplemented mice compared with the HF mice (*P* < 0.05)(Figure 1D). Histological examination of WAT showed that HF+HZ significantly decreased adipocyte size (Figure 1E and 1F). Blood profile measurements showed that HF-induced hypertriglycemia, hyperleptinemia, hyperinsulinemia, hyperglycemia, and increase in FFA and homeostasis model assessment-estimated insulin resistance (HOMA-IR) were all significantly improved following zerumbone treatment (Table 1).

**Table 1:** Effect of zerumbone on serum glucose and lipid profiles

	NC	HF	HF + LZ	HF + HZ
Triglyceride (mg/dL)	48.13 ± 4.07*	53.59 ± 1.74	40.32 ± 1.38*	37.21 ± 2.11*
TC (mg/dL)	81.24 ± 7.15*	128.97 ± 138.08	126.66 ± 7.15	133.99 ± 11.24
HDL-C (mg/dL)	43.56 ± 6.07*	50.30 ± 59.22	60.11 ± 12.79	68.89 ± 6.40*
HDL-C/TC	0.53 ± 0.11*	0.38 ± 0.44	0.46 ± 0.07	0.49 ± 0.04
FFA (µEq/L)	512.40 ± 67.02*	784.30 ± 16.27	601.9 ± 28.35	453.90 ± 7.03*
Leptin (ng/mL)	23.89 ± 7.95*	90.61 ± 45.05	61.52 ± 13.20*	55.47 ± 6.36*
Insulin (ng/mL)	0.30 ± 0.07*	0.60 ± 0.39	0.44 ± 0.11*	0.31 ± 0.05*
Glucose (mg/dL)	264.76 ± 48.29*	501.60 ± 41.18	387.70 ± 23.67*	329.20 ± 30.45*
HOMA-IR	4.89 ± 0.42*	10.39 ± 1.56	6.85 ± 0.67*	6.47 ± 0.39*

Each group consisted of 10 mice. Results are the means ± SEM. *P < 0.05 versus HF group.

NC, normal control diet-fed mice; HF, high-fat diet fed mice; HF+LZ, high-fat diet containing 0.01% zerumbone-fed mice; HF+HZ, high-fat diet containing 0.025% zerumbone-fed mice

TC, total cholesterol; HDL-C, high density lipoprotein-cholesterol; FFA, free fatty acid; HOMA-IR, homeostasis model assessment-estimated insulin resistance.

The alanine transaminase (ALT) and aspartate transaminase (AST) are standard biomarkers for drug-induced liver disease [26]. To evaluate zerumbone-induced hepatotoxicity, we measured the AST/ALT activity in the liver tissues and found that zerumbone did not induce liver toxicity (Figure 1G and 1H).

### Zerumbone ameliorated dysregulated lipid metabolism in WAT

**Figure 2:**
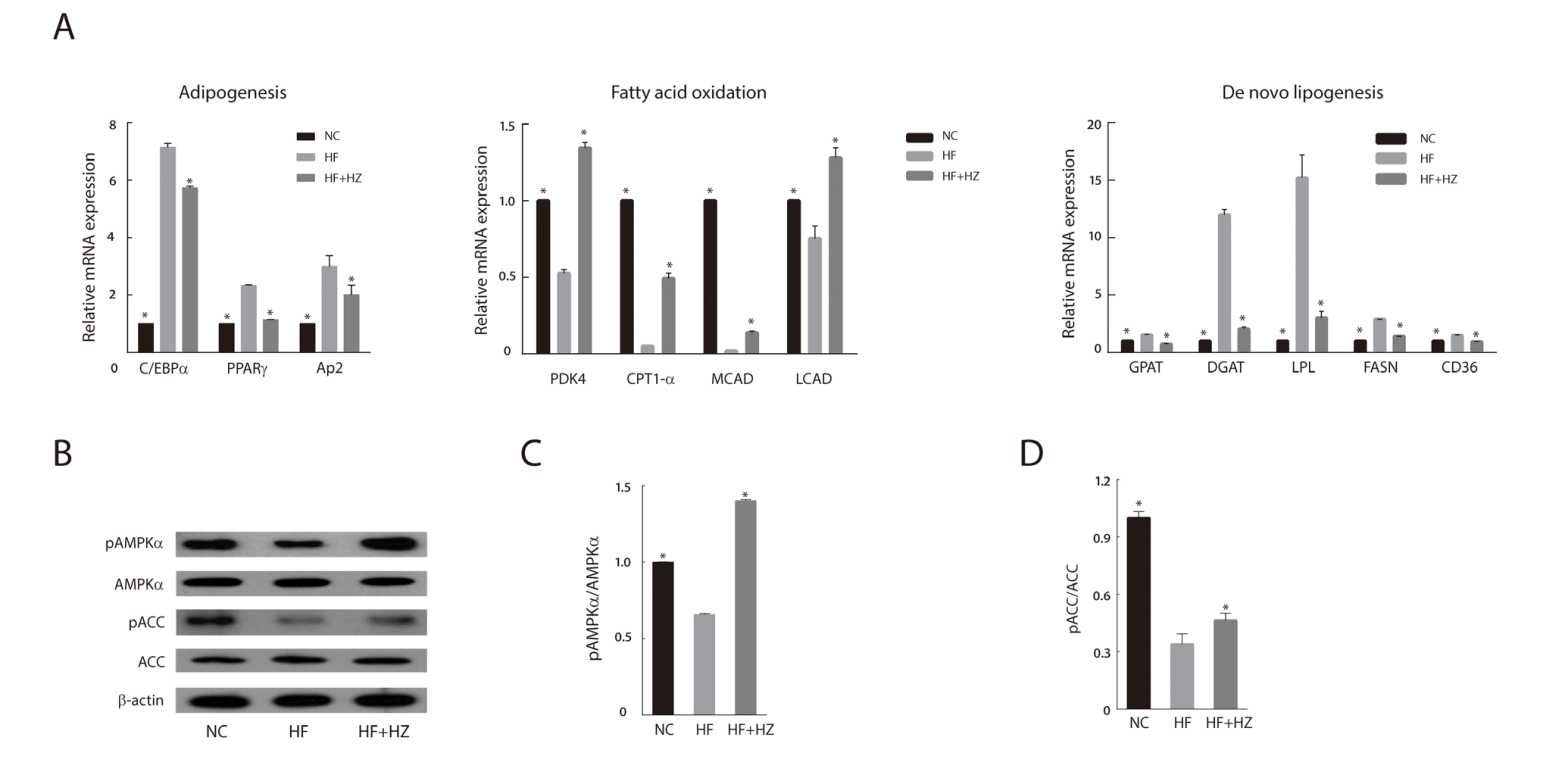
Zerumbone inhibited adipogenesis and modulated lipid metabolism in WAT **A**. Effect of zerumbone on mRNA levels of lipid metabolism-related genes in WAT from mice fed NC, HF, and HF + HZ. **B**. Effect of zerumbone on AMPK signaling in WAT was determined by western blot. **C**. Ratio of phosphorylated AMPK to total AMPK in WAT. **D**. Ratio of phosphorylated ACC to total ACC in WAT. *P < 0.05 versus HF.

We analyzed the effect of zerumbone supplementation on lipid metabolism in WAT. HF feeding altered the lipid metabolism of WAT with upregulation of adipogenic and lipogenic genes, as well as downregulation of fatty acid oxidation-regulating genes (Figure 2A). We observed that zerumbone treatment effectively reversed the changes in the expression of genes involved in lipid metabolism in WAT.

Activation of AMPK in adipose tissue limits fatty acid efflux from adipocytes and favors fatty acid oxidation [27]. In addition, AMPK decreases *de novo* fatty acid synthesis through phosphorylation-mediated inhibition of acetyl-CoA carboxylase (ACC) [28]. Given the important role of AMPK in the regulation of adipose tissue metabolism, we evaluated the effect of zerumbone-induced AMPK activation on adipose metabolism. As shown in Figure 2B, zerumbone increased the phosphorylation of AMPK in WAT; however, the total AMPK content of the experimental groups was similar. The increase in AMPK phosphorylation corresponded with increased ACC (Figure 2C and 2D).

### Zerumbone inhibited adipogenesis in 3T3-L1 cells

**Figure 3:**
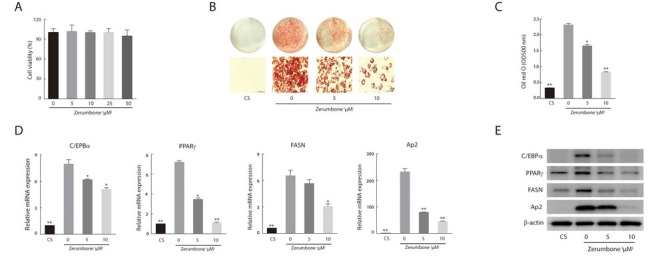
Anti-adipogenic effect of zerumbone in 3T3-L1 cells **A**. Effect of zerumbone on cell viability after 48 h exposure. **B**. Oil Red O staining of 3T3-L1 cells that were differentiated for 10 days. Cells were treated with 5 or 10 μM zerumbone for 48 h at day 0. Non-differentiated preadipocytes (CS) were incubated in DMEM with 10% calf serum. Representative photomicrographs (×200) are shown for each treatment group. **C**. Intracellular lipid accumulation was quantified by measuring optical absorbance at 500 nm. **D**. Real-time PCR analysis of adipogenic transcription factors and their targets in 3T3-L1 cells. The level of each mRNA is expressed relative to the average level of CS. *P < 0.05, **P < 0.01 versus differentiated adipocytes. **E**. Western blotting was performed for various adipocyte differentiation markers.

As zerumbone has been reported to induce apoptotic cell death in several cancer cell lines [29, 30], we tested the cytotoxic effects of zerumbone, and found that it did not exert cytotoxicity up to a concentration of 50 μM (Figure 3A). Zerumbone inhibited adipogenesis in a concentration-dependent manner (Figure 3B and 3C). Differentiation-induced lipid accumulation was accompanied by upregulation of the master adipogenic transcription factors CCAAT enhancer binding protein alpha (C/EBPα) and PPARγ. Zerumbone inhibited the induction of these transcription factors as well as that of fatty acid synthase (FASN, a downstream target of PPARγ) and adipocyte protein 2 (Ap2, a carrier protein expressed in adipocytes) in both mRNA and protein levels (Figure 3D and 3E). These results suggest that zerumbone inhibits adipogenic differentiation via downregulation of C/EBPα and PPARγ.

### Zerumbone reversed the dysregulated miRNA expression in WAT and 3T3-L1 cells

MiRNAs play regulatory roles in obesity by regulating adipocyte differentiation, insulin activity, and fat metabolism [31]. The miRNA expression profiling in obese and lean WAT from mice has revealed that miRNAs are dysregulated in obese adipose tissue [32–34]. Based on this evidence, we selected 21 dysregulated miRNAs and compared their expression in WAT from NC, HF, and HF+Z groups (Table 2). The 10 most upregulated miRNAs were miR-146b, miR-297b, miR-34a, miR-469, miR-139-3p, miR-21, miR-466E-5p, miR22*, miR-324, and miR-143. We additionally measured the expression of these 10 miRNAs in the differentiated 3T3-L1 cells. miR-146b exhibited the highest upregulation, with a 3.39-fold increase in the WAT of HF mice and an 8.12-fold increase in differentiated 3T3-L1 cells. Interestingly, zerumbone treatment effectively reversed the dysregulation of miR-146b expression in WAT from HF and in differentiated 3T3-L1 cells. These results suggest that zerumbone is a negative regulator of miR-146b in WAT from obese mice and in differentiated adipocytes.

**Table 2:** Effect of zerumbone on microRNA expressions in differentiated 3T3-L1 cells and C57BL/6N mice

	HF	HF + Z	D	D+Z
**mmu-miR-146b**	**3.39 ± 0.03**	1.84 ± 0.02*	**8.12 ± 0.85**	**3.43 ± 0.06**^#^
**mmu-miR-297b**	3.18 ± 0.01	1.23 ± 0.01*	0.58 ± 0.01	1.28 ± 0.03^#^
**mmu-miR-34a**	3.14 ± 0.02	2.58 ± 0.06*	6.93 ± 0.34	1.90 ± 0.04^#^
**mmu-miR-469**	2.87 ± 0.03	1.46 ± 0.15*	0.46 ± 0.26	3.35 ± 0.11^#^
**mmu-miR-139-3p**	2.63 ± 0.03	1.20 ± 0.05*	0.03 ± 0.0	0.54 ± 0.02^#^
**mmu-miR-21**	2.50 ± 0.21	2.24 ± 0.2	6.17 ± 0.25	1.06 ± 0.06^#^
**mmu-miR-466E**	2.40 ± 0.01	0.98 ± 0.01*	0.57 ± 0.01	1.07 ± 0.04^#^
mmu-miR-22*	2.18 ± 0.01	1.68 ± 0.20*	0.35 ± 0.02	0.53 ± 0.00^#^
**mmu-miR-324**	2.08 ± 0.01	1.53 ± 0.01*	0.32 ± 0.01	0.51 ± 0.03^#^
**mmu-miR-143**	1.93 ± 0.03	1.53 ± 0.07*	1.27 ± 0.02	0.95 ± 0.01^#^
**mmu-miR-720**	1.59 ± 0.03	1.29 ± 0.05*		
**mmu-miR-15b**	1.46 ± 0.04	0.56 ± 0.03*		
**mmu-miR-302a**	1.42 ± 0.01	0.92 ± 0.03*		
**mmu-miR-466h**	1.34 ± 0.04	1.48 ± 0.06*		
**mmu-miR-197**	1.31 ± 0.01	1.09 ± 0.05*		
**mmu-miR-423**	1.27 ± 0.05	0.96 ± 0.05*		
**mmu-miR-715**	1.25 ± 0.01	1.31 ± 0.16*		
**mmu-miR-467**	1.24 ± 0.00	1.63 ± 0.1*		
**mmu-miR-709**	0.97 ± 0.01	0.73 ± 0.03*		
**mmu-miR-122**	0.69 ± 0.04	0.05 ± 0.06*		
**mmu-miR-802**	0.37 ± 0.01	2.76 ± 0.10*		

Results are the means ± SD. For epididymal adipose tissue, expressions of miRNAs are shown as fold-change relative to the level of NC group. *P < 0.05 versus HF group. For 3T3-cells, expressions of miRNAs are shown as fold-change relative to the level of non-differentiated preadipocytes. ^#^*P* < 0.05 versus D group.

HF, high-fat diet-fed mice; HF+Z, high-fat diet containing 0.025% zerumbone-fed mice; D, differentiated adipocytes; D+Z, differentiated adipocytes treated with10 μM zerumbone.

### Anti-adipogenic effect of zerumbone is dependent on SIRT1, a direct target of miR-146b

**Figure 4:**
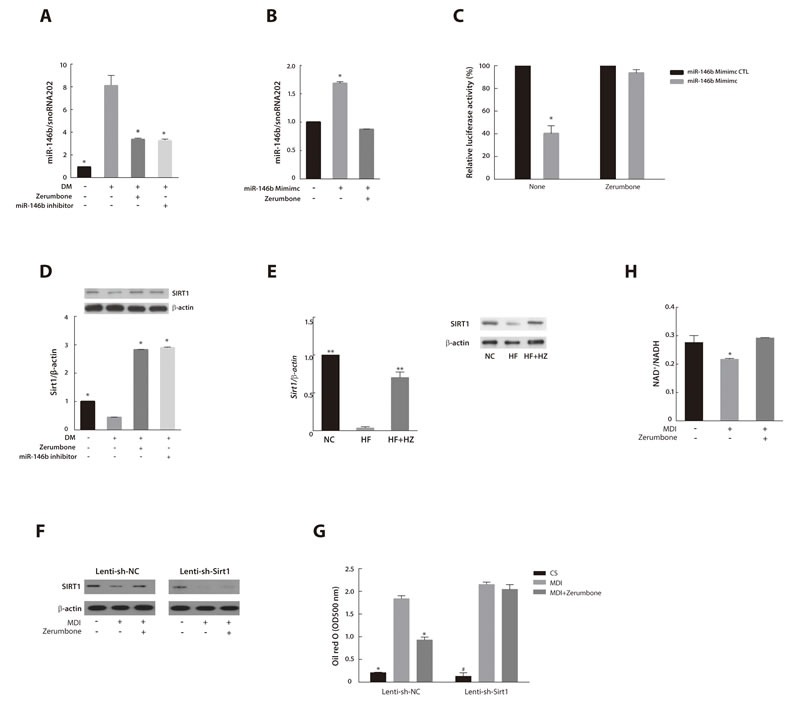
MiR-146b/SIRT1 pathway mediated the anti-adipogenic effect of zerumbone **A**. Effects of zerumbone and miR-146b inhibitor on miR-146b expression in differentiated 3T3-L1 cells. 3T3-L1 cells were differentiated in the presence of zerumbone (10 μM) or miR-146b inhibitor (10 nM). *P < 0.05 versus DM. **B**. Effect of zerumbone on increased miR-146b expression evoked by treatment with the miR-146b mimic. 3T3-L1 cells were incubated in the presence of or miR-146b activator (10 nM) or zerumbone (10 μM) at day 3 for 48 h. *P < 0.05 versus control. **C**. Effect of zerumbone on binding of miR-146b to the 3′-UTR of SIRT1 in 3T3-L1 cells. *P < 0.05 versus miR-146b mimic control with no treatment. **D**. Effect of zerumbone on SIRT1 mRNA and protein expression in differentiated 3T3-L1 cells. *P < 0.05 versus DM. **E**. Effect of zerumbone on SIRT1 mRNA and protein expressions in WAT from mice fed NC, HF, and HF+HZ. **P < 0.01 versus HF. **F**. SIRT1 protein expression was measured by western blot. Preadipocytes transduced with Lenti-sh-NC or Lenti-sh-Sirt1 were differentiated in the presence of 10 μM zerumbone at day 3 for 48 h. **G**. Intracellular lipid accumulation was detected by Oil Red O staining and quantified spectrophotometrically in Lenti-sh-NC- or Lenti-sh-Sirt1-transduced 3T3-L1 cells. *P < 0.05 versus differentiated Lenti-sh-NC, # *P* < 0.05 versus differentiated Lenti-sh-Sirt1. **H**. Effect of zerumbone on NAD^+^/NADH ratio in differentiated 3T3-L1 cells. *P < 0.05 versus control.

To evaluate the effect of zerumbone on miR-146b expression, we treated fully differentiated 3T3-L1 cells with zerumbone or miR-146b inhibitor. Zerumbone inhibited miR-146b expression similar to that observed with miR-146b inhibitor treatment (Figure 4A). Conversely, when 3T3-L1 cells were exposed to a miR-146b mimic and zerumbone simultaneously, zerumbone inhibited the upregulation of miR-146b caused by the miR-146b mimic (Figure 4B). miRNA induces repression of protein translocation or degradation of mRNA through imperfect complementary binding between the miRNA and target mRNA [35]. Therefore, we investigated the effect of zerumbone on the binding activity of miR-146b. Previously, we reported that miR-146b acts as a negative regulator of SIRT1 during adipogenesis [25]. Zerumbone interfered with the binding of miR-146b to the seed sequence of SIRT1 and thus reduced SIRT1 promoter-derived luciferase activity (Figure 4C)

To investigate the mechanisms by which the downregulation of miR-146b by zerumbone affects SIRT1 and the adipogenic response, we measured the SIRT1 mRNA expression and protein levels in the zerumbone-treated or miR-146b inhibitor-treated 3T3-L1 cells. The downregulation of SIRT1 in differentiated 3T3-L1 cells was markedly reversed by treatment with zerumbone and the miR-146b inhibitor (*P* < 0.05) (Figure 4D). The decrease in SIRT1 levels in WAT of HF-fed mice was also attenuated by zerumbone treatment (Figure 4E).

To determine whether the anti-adipogenic effect of zerumbone is mediated by SIRT1, we treated SIRT1-knockdown 3T3-L1 cells, generated using a lentiviral-based SIRT1 shRNA (Lenti-sh-Sirt1), with zerumbone (Figure 4F). Zerumbone failed to inhibit adipogenesis in SIRT1-knockdown 3T3-L1 cells (Figure 4G).

SIRT1 is a NAD^+^-dependent deacetylase that matches the energy output to the energy requirement in the cell by sensing changes in intracellular NAD^+^ [36]. The NAD biosynthesis pathway is mediated by nicotinamide phosphoribosyltransferase (NAMPT), the rate-limiting enzyme for nicotinamide mononucleotide [37]. Zerumbone was also found to augment SIRT1 activity by increasing the NAD^+^/NADH ratio (Figure 4H). Additionally, zerumbone upregulated NAMPT mRNA in 3T3-L1 adipocytes (Figure S1). These results indicate that SIRT1 mediates the anti-adipogenic effect of zerumbone.

### Zerumbone increased deacetylation of FOXO1 and PGG1-α

**Figure 5:**
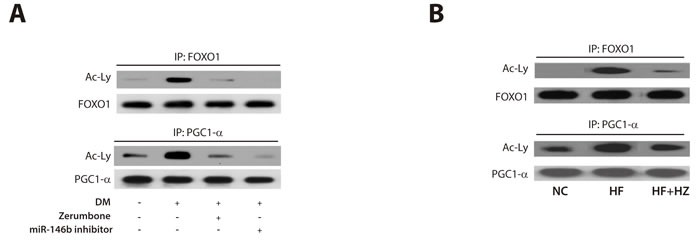
Zerumbone increased deacetylation of FOXO1 and PGC1-α Immunoprecipitation was performed to measure the effect of zerumbone on acetylation of the SIRT1 targets, FOXO1 and PGC1-α, in differentiated 3T3- L1 cells **A**. and WAT from mice **B**.

SIRT1 promotes gene transcription by deacetylating specific transcription factors such as forkhead box O 1 (FOXO1) [38] and peroxisome proliferator-activated receptor gamma coactivator 1-alpha (PGC1-α) [39]. To further investigate the mechanism by which zerumbone regulates the miR-146b/SIRT1 axis to reduce obesity and adipogenesis, we analyzed its effect on the acetylation of FOXO1 and PGC1-α, two downstream targets of SIRT1. As shown in Figure 5A, adipogenic stimulation increased the acetylation of FOXO1 and PGC1-α. This increase was clearly inhibited following treatment with zerumbone and the miR-146b inhibitor. Similarly, the HF feeding-induced increase in the acetylation of FOXO1 and PGC1-α was markedly reduced by zerumbone in WAT (Figure 5B). These data support the idea that SIRT1 regulates the action of zerumbone via deacetylation of FOXO1 and PGC1-α.

## DISCUSSION

**Figure 6:**
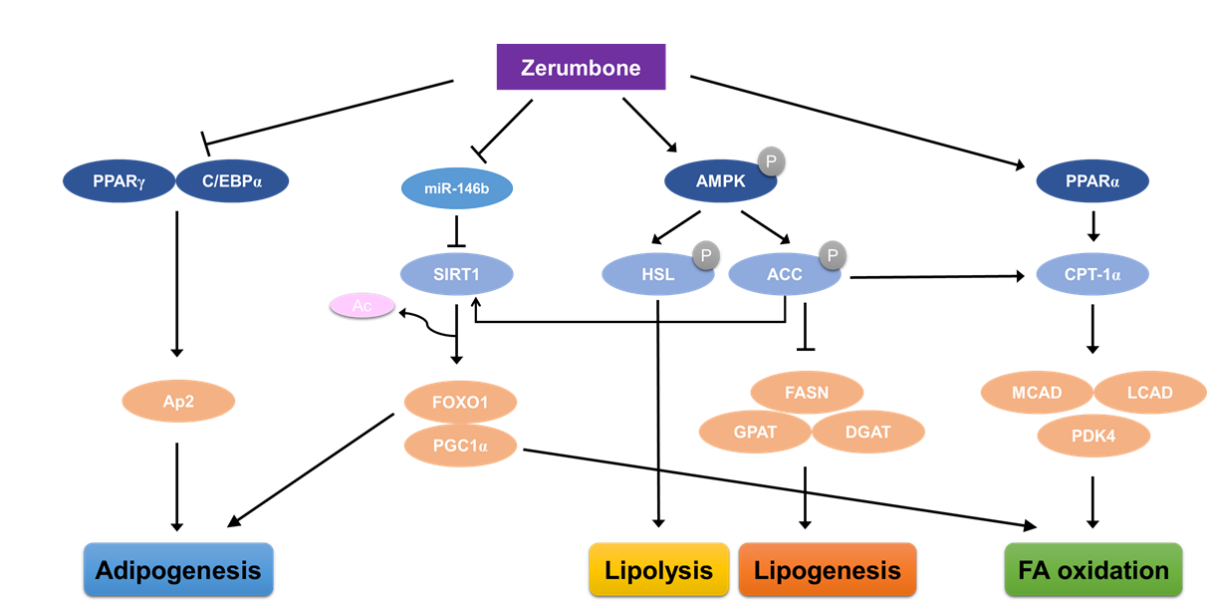
Proposed mechanism through which zerumbone improves diet-induced adiposity in WAT via the AMPK and miR-146b/SIRT1 pathways Zerumbone induces phosphorylation of AMPK and, following phosphorylation of ACC, induces increased fatty acid oxidation and reduced lipogenesis. Zerumbone also acts as a miR-146b inhibitor and downregulates miR-146b, leading to SIRT1 activation. This results in the deacetylation of the SIRT1 targets, FOXO1 and PGC1-α, and improves adiposity by increasing fat mobilization and improving lipid metabolism.

In the present study, we showed that zerumbone protected mice from HF-induced obesity and improved impaired lipid metabolism in WAT via AMPK phosphorylation. Zerumbone also ameliorated dysregulated miRNA profiles in obese WAT tissues and in mature 3T3-L1 cells, with the most prominent change observed in the expression of miR-146b. Zerumbone acts as a miR-146b inhibitor, increasing SIRT1 expression followed by deacetylation of FOXO1 and PGC1-α. The proposed mechanism underlying the amelioration of HF-evoked metabolic dysfunction by zerumbone in WAT is summarized in Figure 6.

We previously reported that zerumbone mitigates high-fat diet-induced obesogenic effects [25]. However, the exact molecular mechanism by which zerumbone alleviates metabolic disorders has not been reported to date. In this study, we found that zerumbone prevented the development of diet-induced adiposity and inhibited 3T3-L1 differentiation. Zerumbone induced AMPK phosphorylation and decreased adiposity-related upregulation of miR-146b. Thus, zerumbone induced AMPK activation in WAT and improved lipid metabolism through upregulation of fatty oxidation-regulated genes and downregulation of lipogenesis-related genes.

SIRT1 activation results in beneficial effects in metabolic disorders involving obesity, type 2 diabetes, and cardiovascular disease [40]. Therefore, activating SIRT1 pharmacologically should be beneficial in treating obesity and age-related diseases. Regulation of SIRT1 by miRNA has been suggested [41]. Downregulation of miR-34a has been shown to increase the expression of the fibroblast growth factor 21 receptor complex and SIRT1, contributing to the activation of browning in adipose tissue [42]. MiR-34a also reduces NAD^+^ levels and SIRT1 activity by directing targeting NAMPT [43]. In recent reports, miR-3a has been reported to target SIRT1 to inhibit prostate cancer cell proliferation [44]. Furthermore, miR-212 negatively regulates autophagy by inhibiting SIRT1 [45]. In addition, we previously reported that miR-146b promotes adipogenesis and obesity by downregulating SIRT1 [25]. In this study, we observed a decrease in miR-146b levels and a subsequent increase in SIRT1 expression in zerumbone-treated 3T3-L1 cells. Furthermore, the anti-adipogenic effect of zerumbone was abolished in 3T3-L1 cells with SIRT1 knockdown, suggesting that zerumbone induced SIRT1 expression by downregulating miR-146b and increasing the NAD^+^/NADH ratio. Upregulation of NAMPT by zerumbone was also observed in adipocytes. Zerumbone supplementation increased SIRT1 activity, ameliorated HF-induced obesity, and reduced HF-induced WAT hypertrophy and hyperplasia in C57BL/6N mice. We additionally observed miR-146b downregulation and subsequent SIRT1 upregulation in the WAT of zerumbone-supplemented mice. Increased SIRT1 expression has additionally been observed in the adipose tissue of obese patients following weight loss [46].

SIRT1 regulates gene transcription by deacetylating specific transcription factors, including FOXO1 [47] and PGC1-α [48]. SIRT1-induced deacetylation of FOXO1 also decreases the expression of adipogenesis-related genes [25]. We found that zerumbone deacetylated FOXO1, which led to downregulation of adipogenesis-related genes in adipose tissue. SIRT1 catalyzes deacetylation of PGC1α, leading to induction of uncoupling protein 1 (UCP1), which is involved in thermogenesis regulation [49].

The miR-146b has been reported to serve as a prognostic marker for non-small lung cancer [50]. The miR-146b inhibits metastasis in glioma [51] and breast cancer cells [52], rescues hypoxia-induced apoptosis in cardiomyocytes [53], and ameliorates retinal inflammation in diabetes [54]. Previously, we demonstrated that inhibition of miR-146b alleviates diet-induced obesity through SIRT1 regulation. In this study, we attempted to identify a new compound involved in the miR-146b/SIRT1 pathway, and found that zerumbone is a candidate for prevention of obesity.

In summary, we showed that zerumbone exerts protective effects against HF-induced obesity and inhibits adipocyte differentiation. Moreover, zerumbone improves impaired lipid metabolism by increasing fatty acid oxidation and reducing lipogenesis via AMPK phosphorylation. The downregulation of miR-146b observed following zerumbone treatment resulted in increased mRNA and protein expression of SIRT1. The consequent deacetylation of FOXO1 and PGC1-α resulted in reduced adipogenesis and inhibited weight gain. These results indicate that zerumbone may serve as a potential therapeutic agent by inhibiting adipogenesis and protecting against high-fat diet induced obesity.

## MATERIALS AND METHODS

### Animals

Five-week-old male C57BL/6N mice were obtained from Orient Bio Inc. (Seongnam, Korea). Animals were housed at a temperature of 24 ± 2°C in 50-60% relative humidity, with 12 h light/dark cycles throughout the experiment. Water and food were supplied *ad libitum*. After 1 week of acclimation, the mice were randomly divided into four groups (*n* = 10 per group): (1) NC (10% of total calories from fat), (2) HF (45% of total calories from fat), (3) HF containing 0.01% zerumbone (HF + LZ), and (4) HF containing 0.025% zerumbone (HF + HZ). The composition of the experimental diets is shown in Table S1. All diets were based on the AIN-76A diet [55]. Body weights were recorded weekly. After 8 weeks on the diet, the mice were euthanized by cervical dislocation. All animal experiments in this study were conducted in accordance with a protocol approved by the Korea Food Research Institute-Institutional Animal Care and Use Committee (KFRI-IACUC No. 2013-149).

### Histological examination

Tissues were fixed in 10% buffered formalin, embedded in paraffin, sectioned, and stained with hematoxylin and eosin (H&E) for histological analyses. The stained areas were observed using a light microscope (Olympus, Tokyo, Japan) with a magnification of 200 ×. The mean adipocyte size was quantified using ImageJ software (NIH, Bethesda, MD, USA).

### Serum and liver biochemistry

Serum glucose, triglyceride (TG), total cholesterol (TC), high-density lipoprotein (HDL), and free fatty acid (FFA) levels were measured enzymatically as previously described [56]. Serum insulin and leptin levels were measured using an ELISA kit (ALPCO Diagnostics, Salem, NH). To measure hepatotoxicity, the AST and ALT activity of liver homogenates was measured.

### Quantitative real-time polymerase chain reaction

Total RNA was isolated using a NucleoSpin RNA II kit (Macherey-Nagel, Duren, Germany). For miRNA measurement, total RNA was reverse transcribed using the TaqMan MicroRNA reverse transcription kit and analyzed by real-time PCR using the TaqMan MicroRNA assay kit (Applied Biosystems, Foster City, CA, USA). Expression of miRNA was normalized to that of endogenous snoRNA202. For the mRNA expression assay, qRT-PCR was performed using SYB Green PCR Master Mix in a StepOnePlus Real-Time PCR system (Applied Biosystems). The level of each mRNA was normalized to that of β-actin. The oligonucleotide sequences for each primer are listed in S2 Table.

### Western blot

Cells and WAT were lysed with RIPA buffer. Western blot analysis was performed as previously described [57]. Blots were probed with primary antibodies against PGC1-α, C/EBPα, FASN, Ap2 (Cell Signaling, Danvers, MA), PPARγ, SIRT1, FOXO1, β-actin (Santa Cruz Biotechnology Inc., Santa Cruz, CA).

### Cell culture and differentiation

3T3-L1 fibroblasts (ATCC, Manassas, VA) or transduced cells were maintained and differentiated as described previously [57]. Briefly, 3T3-L1 cells were maintained in Dulbecco's modified Eagle medium (DMEM) containing 25 mM glucose, 10% calf serum, 100 U/mL penicillin, 100 μg/mL streptomycin, and 4 mM L-glutamine. The cytotoxic effects of zerumbone (> 98%, Z3902, Sigma Aldrich, St. Louis, MO) against 3T3-L1 cells were determined using the 3-[4,5-dimethylthiazol-2-yl]-2,5 diphenyl tetrazolium bromide (MTT) assay as previously described [58]. Following differentiation, cells were stained with Oil Red O (Sigma-Aldrich), dissolved in isopropanol, and quantified by measuring the optical absorbance at 500 nm. Images were collected using an Olympus microscope at a magnification of ×100 (Tokyo, Japan). To induce SIRT1 knockdown, 3T3-L1 cells were transduced with a lentiviral-based SIRT1 short hairpin RNA (shRNA; Genepharma, Shanghai, China). SIRT1 knockdown was confirmed by immunoblotting with an anti-SIRT1 antibody.

### Functional study of miRNA

To modulate miR-146b expression, we transfected miR-146b-specific inhibitor or activator into 3T3-L1 cells, as previously described [25]. Oligonucleotides (10 nM) were transfected into cells with Lipofectamine RNAiMAX (Invitrogen, Carlsbad, CA). Overexpression or inhibition of miR-146b expression was verified by qRT-PCR. The effect of zerumbone on binding efficacy between miR-146b and its target was measured using 3′-UTR luciferase reporter assays, as previously reported by Ahn et al. [25]. Luciferase activity was normalized to the corresponding β-galactosidase activity.

### NAD^+^/NADH ratio measurement

The NAD^+^/NADH ratio was measured in whole-cell extracts of 3T3-L1 cells using a NAD^+^/NADH quantification kit (Biovision, San Francisco, CA, USA). The absorbance was measured at 450 nm and normalized to protein the concentration.

### Immunoprecipitation

Immunoprecipitation was performed using the protein A immunoprecipitation kit (Roche Applied Science, Mannheim, Germany) by mixing cell lysates with an antibody against FOXO1 and PGC1-α. Immunoprecipitated proteins were separated by gel electrophoresis and subjected to western blotting with an antibody against acetylated lysine (Santa Cruz Biotechnology).

### Statistical analysis

Results were expressed as the mean ± standard deviation (SD). Statistical analyses were performed using GraphPad Prism 6 software (San Diego, CA). One-way analysis of variance (ANOVA) was used to compare quantitative data among groups. The Bonferroni post-hoc test was used if ANOVA indicated significance (*P* < 0.05).

## SUPPLEMENTARY MATERIALS FIGURES AND TABLES



## Abbreviations

C/EBPα: CCAAT enhancer binding protein alpha
FOXO1: forkhead box O 1
HF: high-fat diet
miRNA: microRNA
PGC-1α: peroxisome proliferator-activated receptor gamma coactivator 1 alpha
SIRT1: sirtuin 1
WAT: white adipose tissue.
