# Supplementary material for: Zerumbone ameliorates high-fat diet-induced adiposity by restoring AMPK-regulated lipogenesis and microRNA-146b/SIRT1-mediated adipogenesis
Source: Oncotarget. 2017 Apr 8;8(23):36984–95. doi: 10.18632/oncotarget.16974 (PMC5514886; doi:10.18632/oncotarget.16974)
Supplement: Supplementary file 1 [file oncotarget-08-36984-s001.pdf]

Zerumbone ameliorates high-fat diet-induced adiposity by restoring AMPK-regulated lipogenesis and microRNA-146b/SIRT1-mediated adipogenesis

Supplementary Material

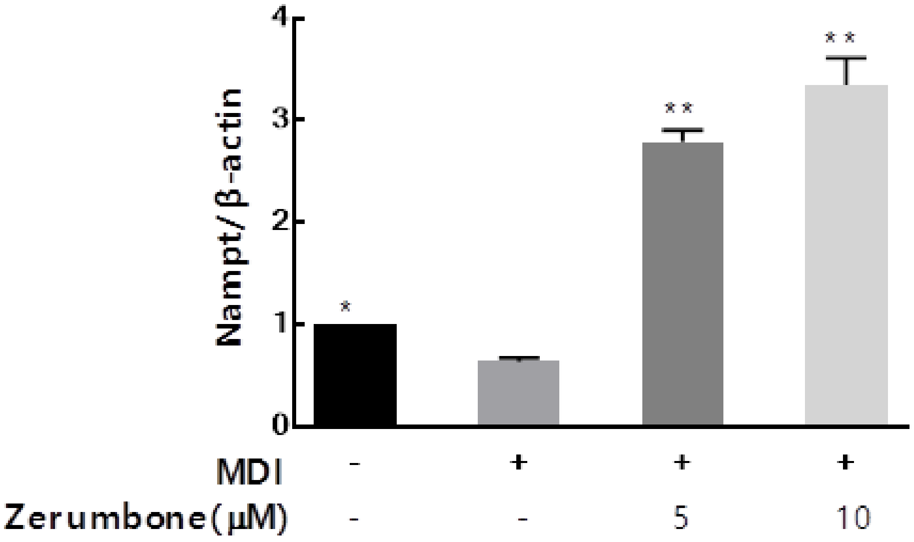

Supplementary Figure 1: Effect of zerumbone on NANMP mRNA levels. 3T3-L1 cells were treated with 5 or 10 μM zerumbone for 48 h at day 0 and differentiated.

Supplementary Table 1: Diet compositions

Component (g)	NC	HF	HF+ZL	HF+ZH
**Casein**	200	200	200	200
**Corn oil**	50	50	50	50
**Lard**	-	50	50	50
**Cholesterol**	-	5	5	5
**Cocoa butter**	-	70	70	70
**Coconut oil**	-	30	30	30
**Corn starch**	350	195	195	195
**Sucrose**	300	300	300	300
**Cellulose**	50	50	50	50
**Mineral mix**	35	35	35	35
**Vitamin mix**	10	10	10	10
**Methionine**	3	3	3	3
**Choline bitartrate**	2	2	2	2
**Zerumbone**	-	-	0.1	0.25

Supplementary Table 2: The primers and reaction conditions.

Gene	NCBI accession number	Primer (5′→3′)
C/EBPα	NM_007678.3	F : CCTTCAACGACGAGTTCCTG
		R : TGGCCTTCTCCTGCTGTC
PPARγ	NM_001127330.1	F : CTGGCCTCCCTGATGAATAA
		R : GGCGGTCTCCACTGAGAATA
Ap2	NM_024406.2	F : CCGCAGACGACAGGA
		R : CTCATGCCCTTTCATAAACT
PDK4	NM_013743.2	F : AGGGAGGTCGAGCTGTTCTC
		R : GGAGTGTTCACTAAGCGGTCA
CPT-1 α	NM_013495.2	F : CTCCGCCTGAGCCATGAAG
		R : CACCAGTGATGATGCCATTCT
MCAD	NM_007382.5	F : GGCAAATGCCTGTGATTCTT
		R : ACCCATTGCGATCTTGAAAC
LCAD	NM_172678.3	F : TCCAGAGGTCAGTCAACATGA
		R : CCTGGTCAATTTTTCGAGAGTCC
GPAT	NM_008149.3	F : CCTTCCATTTCAGTGTTGCAGA
		R : ACAGTTGGCACAATAGACGTTT
DGAT	NM_026384.3	F : AGTGGCAATGCTATCATCATCGT
		R : AAGGAATAAGTGGGAACCAGA TCA
LPL	NM_008509.2	F : GGGAGTTTGGCTCCAGAGTTT
		R : TGTGTCTTCAGGGGTCCTTAG
FASN	NM_007988.3	F : GGAGGTGGTGATAGCGGCTAT
		R : TGGGTAATCCATAGAGCCCAG
CD36	NM_001159555.1	F : GATGACGTGGCAAAGAACAG
		R : AAAGGAGGCTGCGTCTGTG
NAMPT	NM_021524.2	F : CCCGATTGAAGTAAAGGCTGT
		R : TGGTAAGCCAGTAGCACTCTG

Ap2, Adipocyte protein 2; C/EBPα, CCAAT enhancer binding protein alpha; PPARγ, peroxisome proliferator-activated receptor gamma; FASN, fatty acid synthase; LPL, lipoprotein lipase; CD36, Cluster of Differentiation 36; MCAD, medium-chain acyl-CoA dehydrogenase; LCAD, long-chain acyl-CoA dehydrogenase; PDK4, pyruvate dehydrogenase kinase, isozyme 4; GPAT, glycerol-3-phosphate acyltransferase; DGAT, diacylglycerol acyltransferase; CPT-1 α, carnitine palmitoyltransferase-1 alpha
